# Functional and Genomic Analysis of *Rouxiella badensis* SER3 as a Novel Biocontrol Agent of Fungal Pathogens

**DOI:** 10.3389/fmicb.2021.709855

**Published:** 2021-08-05

**Authors:** Luzmaria R. Morales-Cedeño, Sergio de los Santos-Villalobos, Gustavo Santoyo

**Affiliations:** ^1^Instituto de Investigaciones Químico-Biológicas, Universidad Michoacana de San Nicolás de Hidalgo, Morelia, Mexico; ^2^Instituto Tecnológico de Sonora, Ciudad Obregón, Mexico

**Keywords:** genomic analysis, sustainable agriculture, fungal antagonism, postharvest disease, volatile organic compound

## Abstract

In recent decades, various bacterial species have been characterized as biocontrol agents for plant crop diseases; however, only a few genera have been predominantly reported in the literature. Therefore, the identification of new antagonists against phytopathogens is essential for boosting sustainable food production systems. In this study, we evaluated the role of strain SER3 from the recently discovered *Rouxiella badensis* as a biocontrol agent. SER3 was isolated from the phyllosphere of decaying strawberry fruit (*Fragaria* × *ananassa*) and showed different grades of antagonism against 20 fungal pathogens of berries, based on confrontation assays, due to the action of its diffusible and volatile compounds. These fungal pathogens were isolated from decayed strawberry, blackberry, and blueberry fruit and were characterized through internal transcribed spacer (ITS) sequencing and homology searches, exhibiting similarity with well-known postharvest pathogens such as *Botrytis*, *Fusarium*, *Geotrichum*, *Mucor*, *Penicillium*, *Alternaria*, and *Botryosphaeria*. Koch’s postulates were confirmed for most pathogens by reinfecting berry fruit. SER3 showed good capacity to inhibit the growth of *Botrytis cinerea* and *Fusarium brachygibbosum* in strawberry fruit, affecting mycelial development. To gain better understanding of the genetic and metabolic capacities of the SER3 strain, its draft genome was determined and was found to comprise a single chromosome of 5.08 Mb, 52.8% G + C content, and 4,545 protein-coding genes. Phylogenetic analysis indicated that the SER3 strain is affiliated with the *R. badensis* species, with an average nucleotide identity >96% and a genome-to-genome distance >70%. A comparison of the genomic properties of *R. badensis* SER3 and other close bacterial relatives showed several genes with potential functions in biocontrol activities, such as those encoding siderophores, non-ribosomal peptide synthetases, and polyketide synthases. This is the first study to demonstrate a novel role of the recently discovered *R. badensis* species (and any other species of the genus *Rouxiella*) as a biocontrol agent against postharvest fungal pathogens.

## Introduction

The demand for food is increasing worldwide, resulting in the requirement to produce it under eco-friendly systems to ensure food security (Allen and Prosperi, 2016). However, constant attack by fungal and oomycete phytopathogens reduces the yield and quality of crops, causing huge losses at different stages of the agricultural cycle (Dean et al., 2012; Kamoun et al., 2015). For example, *Botrytis cinerea* has been reported to infect more than 200 plant species and cause losses of more than €1 billion/annum globally (Romanazzi and Feliziani, 2014). Similarly, several species of the genus *Fusarium*, together with *Botrytis*, are among the top 10 pathogens worldwide that can cause serious yield losses in agriculture (Magan et al., 2010; Dean et al., 2012). Likewise, invasion by non-native species of phytopathogens owing to transportation and storage of vegetables and fruit is another factor that affects products postharvest (Fried et al., 2017).

Thus, an efficient alternative against crop infestation, which includes the use of antagonistic biological agents, has been developed to eliminate or reduce the use of pesticides in agriculture (Compant et al., 2005; Backer et al., 2018). One of the advantages of biological agents such as *Trichoderma* or bacteria is that they are safe and environment friendly (Elad, 2000; Wang et al., 2020). This group of beneficial microorganisms associated with plants has emerged as a viable, economical, and efficient alternative to control various pre- and postharvest diseases (Morales-Cedeño et al., 2021). Even antagonism in plant growth-promoting bacteria (PGPB) toward phytopathogens is considered an indirect mechanism to stimulate plant growth (Glick, 2012). Their mechanisms of antifungal action against fungal pathogens include the production of diffusible compounds [e.g., hydrolytic enzymes, siderophores, lipopeptides, phenazines, 1-aminocyclopropane-1-carboxylate (ACC) deaminase] or antibiotics and volatiles compounds (e.g., dimethyl disulfide, hydrogen cyanide, and others) (Hernández-León et al., 2015; Khan et al., 2018; Rojas-Solís et al., 2018). Multiple species of PGPB have been isolated and characterized based on their antagonism toward phytopathogens, including *Pseudomonas* spp. and *Bacillus* spp., among few other genera that are predominantly reported (Höfte and Altier, 2010; Santoyo et al., 2012; Islam et al., 2017). Thus, the search for new antagonistic bacterial species is essential to increase the possibility of developing new biofungicides for commercial application (Córdova-Albores et al., 2020).

In this study, we propose a novel ecological role for *Rouxiella badensis* strain SER3 as an antagonist of postharvest pathogens of berries. *R. badensis*, together with *Rouxiella silvae*, was recently proposed as a new bacterial species by Le Fléche-Matéos et al. (2017). Some genera phylogenetically close to *Rouxiella*, such as *Serratia* and *Rahnella*, have previously been described as antagonists and PGPB. For example, Koo and Cho (2009) isolated and characterized a strain of *Serratia* sp. SY5, which had the ability to stimulate the growth of maize seedlings under stressful conditions. In addition, Sun et al. (2020) observed that *Rahnella aquatilis* strain MEM40, isolated from the rhizosphere of a rice plant, showed plant growth promoter effects and antagonism against phytopathogens such as *Magnaporthe oryzae* and *F. graminearum*. The only report on the genus *Rouxiella* described its role as an inhibitor of human pathogenic bacterial growth, but this strain has not been fully characterized, and its taxonomic assignation remains at the genus level (Nam et al., 2020). The isolation of a possible *R. badensis* strain 70 (among other 43 isolated endophytic strains) as an antagonist of pathogenic bacteria and fungi has also been reported. However, the characterization of strain 70 was based only on a partial 16S rDNA sequence (1,023 bp); thus, elucidation of its taxonomic affiliation requires further analysis (Wang et al., 2019). Herein, we present the isolation and characterization of a novel ecological role of *R. badensis* as a biocontrol agent against 20 fungal phytopathogens of berries (*Fragaria* × *ananassa*, *Vaccinium* spp. var. Biloxi, *Rubus* subgenus *Eubatus*), also isolated and characterized in this study. Furthermore, the *R. badensis* SER3 genome was sequenced to support its taxonomic affiliation and mined for detecting biosynthetic gene clusters that could be involved in its biocontrol capabilities.

## Materials and Methods

### Isolation and Characterization of Postharvest Fungal Pathogens

Endophytic fungal pathogens were isolated from berries, including strawberries (*n* = 33), blackberries (*n* = 36), and blueberries (*n* = 49), which were collected from commercial markets. Berry fruits were surface sterilized according to a previous study (Contreras et al., 2016). Briefly, berries were immersed in 70% ethanol for 30 s, then washed with sodium hypochlorite (NaOCI) solution (2.5% available Cl^–^) for 5 min, and then rinsed with ethanol (70% v/v) for 30 s. Finally, the fruits were washed five times with sterile distilled water. Aliquots of sterile distilled water used in the final rinse were cultured on plates containing nutrient agar (NA) medium (Merck). The plates were examined for bacterial growth after incubation at 28°C for 4 days. The sterilized fruits were used in decaying experiments to isolate potentially endophytic fungal pathogens. Briefly, groups of 5–10 fruits (strawberries, blackberries, and blueberries) were placed in disinfected containers, closed, and kept in the dark at room temperature. Fruit weight and firmness were measured on days 1, 5, and 10 (until the growth of fungal mycelium was detected) with an analytical balance (Benchmark Scientific, Inc., Sayreville, NJ, United States) and a penetrometer (Model GY-1, Hangzhou Scientific Instruments), respectively. Koch’s postulates of fungal endophytes were confirmed for most of the pathogens (except *Trichoderma*) as follows: berry fruits were sterilized as described above, placed inside sterile glass bottles, and inoculated with the spores obtained from each fungal culture (∼1 × 10^5^ spores/ml). Mycelial growth of fungi on the fruit was visualized after 5–10 days. Further characterization was performed to confirm fungal identity.

Genomic DNA was extracted from fungal isolates as per the protocol by Mahuku (2004), followed by polymerase chain reaction analysis to amplify the intergenic spacer (ITS) regions with the following primers: ITS4 (5′-TCCTCCGCTTATTGATATGC-3′) and ITS5 (5′-GGAAGTAAAAGTCGTAACAAGG-3′) (Hernández-León et al., 2015). The amplified ITS regions of each of the 20 fungal isolates were sequenced at Macrogen, Seoul, South Korea. Sequences and most probable taxonomic affiliation were deposited in GenBank, and the accession numbers are shown in Table 1.

**TABLE 1:** Fungal strains isolated from strawberries, blackberries and blueberries, with the closest identity based on the ITS sequence homology searches.

**Strain**	**Closest Genbank species identity**	**Identity (%)**	**Access number**	**Source of isolation**
62BCV	*Botrytis cinerea*	99.8	MN365049.1	Strawberries
62C	*Botrytis* sp.	99.4	MN365050.1	
4BF	*Fusarium brachygibbosum*	99.2	MN365015.1	
HBF	*Fusarium brachygibbosum*	98.3	MN365017.1	
FRB	*Geotrichum candidum*	98.3	MN394447.1	
1BF	*Mucor circinelloides*	99.1	MK880497.1	
22	*Mucor fragilis*	99	MN365051.1	
FRA	*Mucor fragilis*	99	MN364941.1	
1F	*Penicillium crustosum*	96.8	MN080331.1	
230	*Penicillium expansum*	99.6	MN393696.1	
5F	*Penicillium expansum*	99.6	MN080332.1	
4AF	*Trichoderma* sp.	98.8	MN365013.1	
2Z	*Alternaria alternata*	99	MN397936.1	Blackberries
7Z	*Geotrichum phurueaensis*	98	MN397937.1	
1A	*Alternaria alternata*	99.6	MK881030.1	Blueberries
3A	*Alternaria* sp.	99.4	MN393668.1	
4A	*Alternaria alternata*	96.2	MN410562.1	
6A	*Alternaria alternata*	97.3	MN365025.1	
5A	*Botryosphaeria rhodina*	99.4	MN364705.1	
1BOA	*Cladosporium* sp.	98.8	MN364646.1	

### Isolation of SER3 and Confrontation Bioassays

Strain SER3 was isolated from the phyllosphere of strawberry fruit and selected for antagonism against the fungal pathogen *F. brachygibbosum* in a prescreening assay in dual culture (Supplementary Figure 1). The strain was grown at 30°C for 24 h on NA medium and maintained at 4°C.

Fungal antagonism by strain SER3 was evaluated as previously reported for Petri dish-based bioassays (Hernández-León et al., 2015). Briefly, SER3 was streaked onto potato dextrose agar (PDA) plates in a cross shape, and then, four mycelial plugs (6-mm diameter) from each of the 20 fungal isolates were deposited in the center of each quadrant. The plates were incubated in the dark at 30°C, and the mycelial growth diameter was measured on day 6.

The antifungal effects of the volatile compounds produced by strain SER3 were also evaluated in Petri plates. SER3 [100 μl, at ∼1 × 10^6^ colony forming units (CFU)] was inoculated on one side of the divided Petri plates, and in the other sections, mycelial plugs of each studied fungus (6-mm diameter) were inoculated. The inoculated plates were incubated, and mycelial growth was measured as described above. Both experiments were independently performed in triplicate, and the inhibition percentage was calculated using the following formula:% growth inhibition = [(Ac − Ab)/Ac] × 100, where Ac is the control mycelial area, and Ab is the mycelial area under treatment.

### Fungal Growth Inhibition Bioassay on Strawberry Fruit and Microscopy Visualization

The strawberries were washed with running water and subsequently placed in a container with 70% ethanol for 1 min. The ethanol was decanted, and then, the berries were washed with 2.5% sodium hypochlorite for 1 min. This process was repeated three times, and finally, the strawberries were rinsed thrice with sterile deionized water.

Following the above procedure, strawberries were allowed to dry in a laminar flow hood, and an incision of approximately 3 mm length, width, and depth was made on each fruit with the tip of a sterile scalpel. Four treatments were performed, using the following: (i) sterile distilled water as the negative control; (ii) a mycelium plug, 7 mm in diameter, of the phytopathogen *B. cinerea* 62BCV or *F. brachygibbosum* 4BF as a positive control; and (iii) a bacterial suspension of SER3 (100 μl, ∼1 × 10^6^ CFU) and a mycelium plug, 7 mm in diameter, of each phytopathogen; and (iv) the supernatants (100 μl) of strain SER3 obtained from nutrient broth after an overnight culture and the mycelium of the two studied phytopathogens grown for 24 h at 29°C. After treatment, the strawberries were placed in closed sterile plastic containers and maintained at room temperature for 3 days.

For microscopy visualization, strain SER3 was simultaneously striated with phytopathogenic fungi (*B. cinerea* 62BCV or *F. brachygibbosum* 4BF) in separate Petri dishes containing PDA. The bacteria were streaked on the cross-shaped dishes, and a 7-mm portion of the mycelium was deposited in the center of each quadrant, as previously mentioned. Subsequently, a mycelium sample was stained with lactophenol blue and safranin and visualized under a Velab VE-BC3 Plus optical microscope.

### SER3 Genome Sequencing and Analysis

A single colony of strain SER3 was picked from a streaked NA plate (BD Bioxon), which was maintained at 30°C overnight. SER3 genomic DNA was extracted following standard protocols (Mahuku, 2004) and further purified using a Wizard^®^ Genomic DNA Purification Kit (Promega, Fitchburg, WI, United States). The quality and quantity of the extracted DNA were evaluated with agarose gel electrophoresis and using a NanoDrop spectrophotometer (Thermo Scientific, Waltham, MA, United States), respectively. Genomic DNA from SER3 was sequenced commercially (MR DNA, Shallowater, TX, United States) by using the Illumina HiSeq technologies platform (2 × 300 bp). FastQC analysis, version 0.11.5, of the raw reads was employed to perform quality control (Andrews, 2010). Trimmomatic, version 0.32, was used to remove bases of low quality and adapter sequences (Bolger et al., 2014). Genome assembly was performed with contigs obtained through the PATRIC^1^ genome service and SPAdes assembler version 3.10.0 (Bankevich et al., 2012). The draft genome of SER3 was reordered according to the reference genome of *Rahnella aquatilis* KM12 (NCBI project accession number: ASM395610v2). PLACNETw was used to explore the presence of plasmids in the SER3 genome (Vielva et al., 2017).

### Taxonomic Affiliation of Strain SER3

The 16S rRNA gene sequence was obtained from the genome and used in basic local alignment search tool (BLAST) homology searches to assign the possible taxonomic affiliation of strain SER3. After that, a genome-level approach was adopted, employing average nucleotide identity (ANI) > 95–96% (Yoon et al., 2017) and a genome-to-genome distance calculator (GGDC) > 70% (Meier-Kolthoff et al., 2013). This genome-level approach was based on strains having cutoff values for species delimitation established for the 16S rRNA gene (>98.7%) (Chun et al., 2018).

### Phylogenomic Analysis of SER3

Phylogenomic relationships of *R. badensis* SER3 and the bacterial strains with high similarity according to ANI and GGDC values were analyzed using the REALPHY pipeline (Bertels et al., 2014). The neighbor-joining method was used for tree construction, and the nucleotide distance was measured using the Jukes–Cantor model. Furthermore, bootstrap analysis with 1,000 replications was performed.

### Genome Annotation and Mining for Plant Growth-Promoting and Biocontrol Traits

The assembled genome was annotated using the Rapid Annotation of the Subsystem Technology (RAST) server^2^. Genome mining was performed by biosynthetic gene cluster (BGC) prediction using antiSMASH 4.0 (Blin et al., 2017) for *R. badensis* SER3 and other close bacterial genomes and manually inspected from the annotations generated by the RAST server^3^ (Aziz et al., 2008), specifically the RASTtk pipeline.

## Results

### Isolation and Characterization of Postharvest Phytopathogens

**FIGURE 1:**
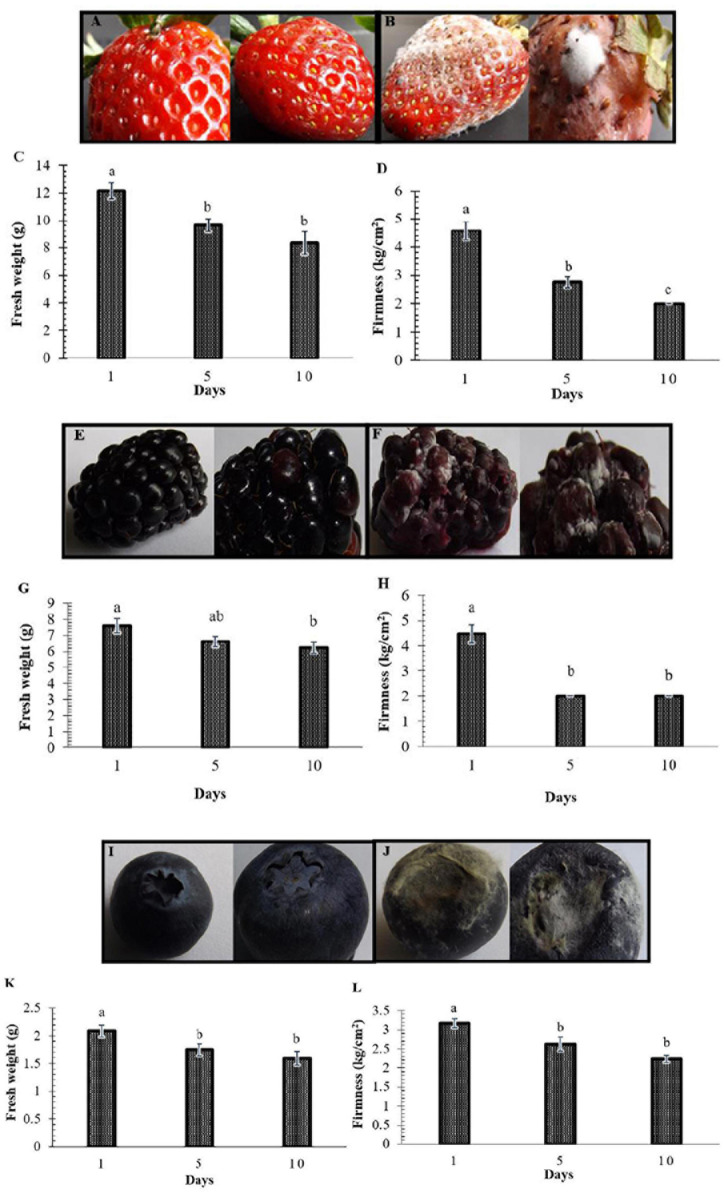
Postharvest decay in berries represented by loss of weight and firmness. Representative fruit are shown in Panels **(A,B,E,F,I,J)**. **(A,E,I)** Fruit at the beginning of the study (control). **(B,F,J)** Fruit in decay on days 5 and 10. Panels **(C,G,K)** show the decrease in the weight of strawberries, blackberries, and blueberries, respectively. Panels **(D,H,L)** show the decline in firmness. Bars represent means ± standard deviation (*n* = 12). The letters indicate that the means differ significantly according to Duncan’s multiple range test (*p* < 0.05).

**FIGURE 2:**
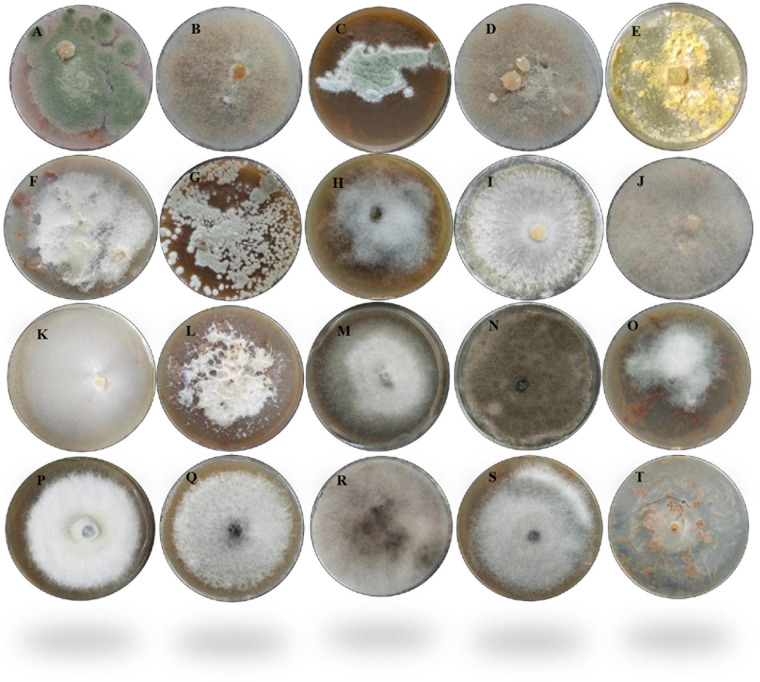
Morphological appearance of the 20 fungal strains isolated from fruit berries. **(A)** *Penicillium crustosum*1F. **(B)** *Mucor circinelloides* 1BF. **(C)** *Penicillium expansum* 230. **(D)** *Mucor fragilis* 22. **(E)** *Trichoderma* sp. 4AF. **(F)** *Fusarium brachygibbosum* 4BF. **(G)** *Penicillium expansum* 5F. **(H)** *Botrytis* sp. 62C. **(I)** *Botrytis cinerea* 62BCV. **(J)** *Mucor fragilis* FRA. **(K)** *Geotrichum candidum* FRB. **(L)** *Fusarium brachygibbosum* HBF. **(M)** *Alternaria alternata* 1A. **(N)** *Cladosporium* sp. 1BOA. **(O)** *Alternaria alternata* 2Z. **(P)** *Alternaria* sp. 3A. **(Q)** *Alternaria alternata* 4A. **(R)** *Botryosphaeria rhodina* 5A. **(S)** *Alternaria alternata* 6A. **(T)** *Geotrichum phurueaensis* 7Z. The fungal growth was observed at different times, ranging from 5 to 8 days.

In this study, the decay of berries over time showed a reduction in fresh weight and fruit firmness between days 5 and 10, consistent with the appearance of decaying symptoms caused by fungal pathogens (Figure 1). Following the decay, 20 berry fungi were isolated. Figure 2 shows the morphological appearance of the isolated fungal strains. Sequencing of the ITS from the isolated fungi showed high homology with *B. cinerea*, *Botrytis* sp., *F. brachygibbosum*, *Geotrichum candidum*, *Geotrichum phurueaesis*, *Mucor circinelloides*, *Mucor fragilis*, *Penicillium crustosum*, *Penicillium expansum*, *Trichoderma* sp., *Alternaria alternata*, *Alternaria* sp., *Botryosphaeria rhodina*, and *Cladosporium* sp. (Table 1). To determine the infection rates of fungi, including those not reported as the main postharvest phytopathogens of berries, Koch’s postulates were confirmed, thus corroborating their role in postharvest fungal infections (Supplementary Figure 2).

### Confrontation Assays

**FIGURE 3:**
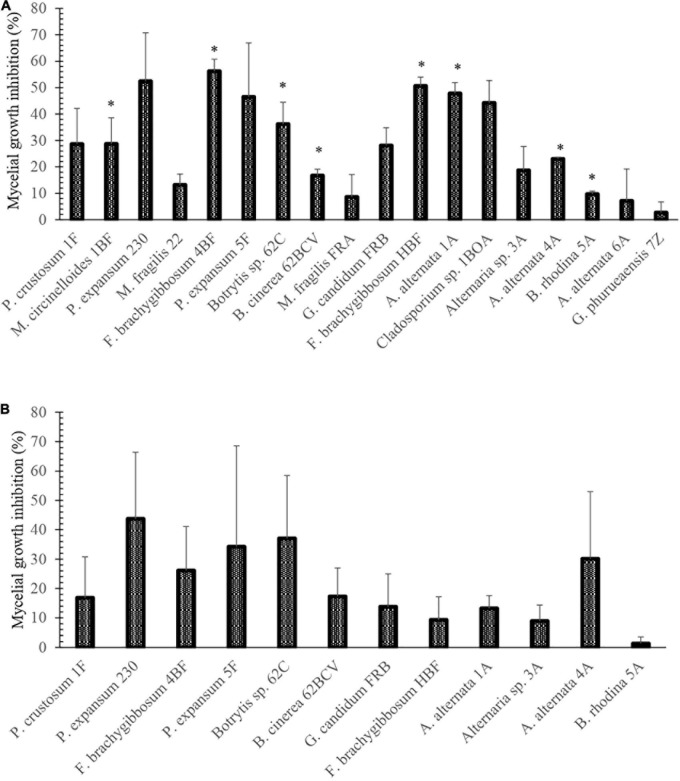
Percentages of inhibition of fungi isolated from berries postharvest caused by **(A)** diffusible and **(B)** volatile compounds of the SER3 strain. The experiments were carried out independently three times. Bars represent mean ± SE. Asterisks indicate significant difference compared to the respective controls, using Student’s *t*-test (*p* < 0.05).

Once the growth and infection capacity of fungal phytopathogens was confirmed in strawberry and blueberry fruit, confrontation tests were performed using strain SER3. SER3 remarkably inhibited mycelial growth through the action of diffusible compounds against eight phytopathogens, such as *Alternaria alternata*, *Botryosphaeria rhodina*, *Mucor circinelloides*, *Botrytis* spp., *and Fusarium* spp. (Figure 3A). Although an inhibitory trend was observed in the growth of some phytopathogens by the action of volatile compounds from SER3, results showed that the inhibition was not significant (Figure 3B).

### *In vivo* Phytopathogen Inhibition Assay Using Strain SER3

**FIGURE 4:**
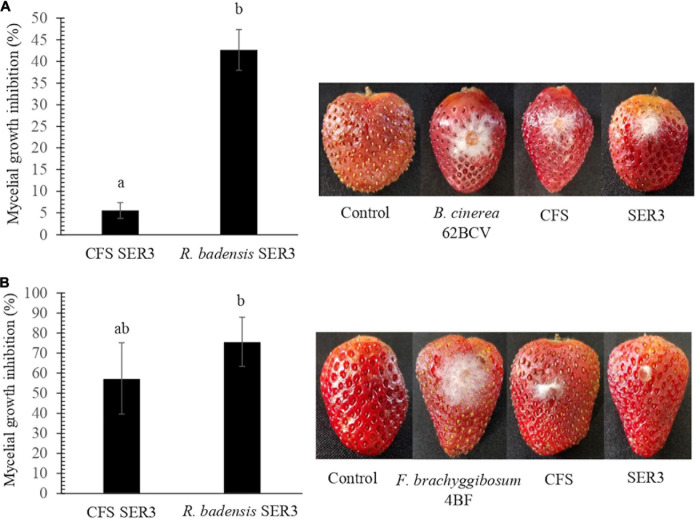
Biocontrol effects of SER3 and its supernatant on strawberries. **(A)** Mycelial growth inhibition of *Botrytis cinerea* by the cell-free supernatant (CFS) and SER3. **(B)** Mycelial growth inhibition of *Fusarium brachyggibosum* by the CFS and SER3. Treatments consisted of sterile distilled water (control) and inoculation with pathogens, CFS from SER3, and cell suspensions of SER3. Experiments were independently performed three times, and bars represent mean ± SE (*n* = 9). Letters indicate significant difference, based on Duncan’s multiple range test (*p* < 0.05).

**FIGURE 5:**
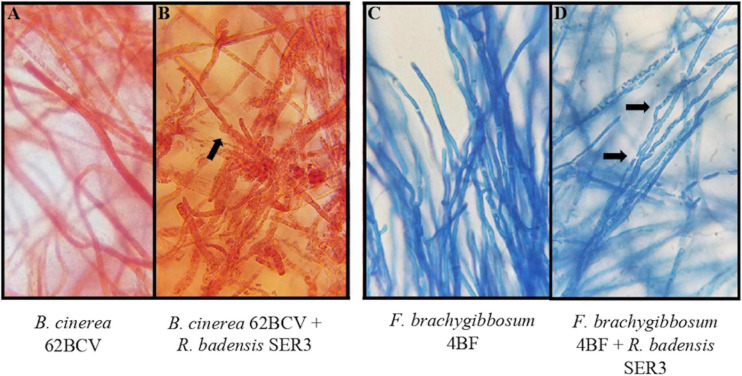
Effect of SER3 on mycelial morphology of **(A,B)** *B. cinerea* and **(C,D)** *F. brachyggibosum*. Panels **(A,C)** represent the controls, Panels **(B,D)** show the interaction between the bacterial strain and each of the pathogens. Arrows indicate distortion of hyphae (×100 magnification).

To evaluate the potential antagonism of strain SER3 against phytopathogens on fruit, two important postharvest phytopathogens (*B. cinerea* 62BCV and *Fusarium brachyggibosum* 4BF) were selected. Figure 4 shows that SER3 produced significant mycelium growth inhibition of *B. cinerea* 62BCV through direct interaction (42.66%), while the cell-free supernatant (CFS) inhibited only 5.55% of phytopathogen growth. With *F. brachyggibosum* 4BF, mycelial growth was inhibited by 75.68%, while CFS restricted mycelial growth by 57.37%. Microscopic analysis of the mycelia of each fungal phytopathogen showed deformations and protrusions in their hyphae on application of the bacterial strain or the cell-free supernatant, whereas typical hyphae were observed in the control in the absence of strain SER3 or its CFS (Figure 5).

### Genome Features of Strain SER3

**FIGURE 6:**
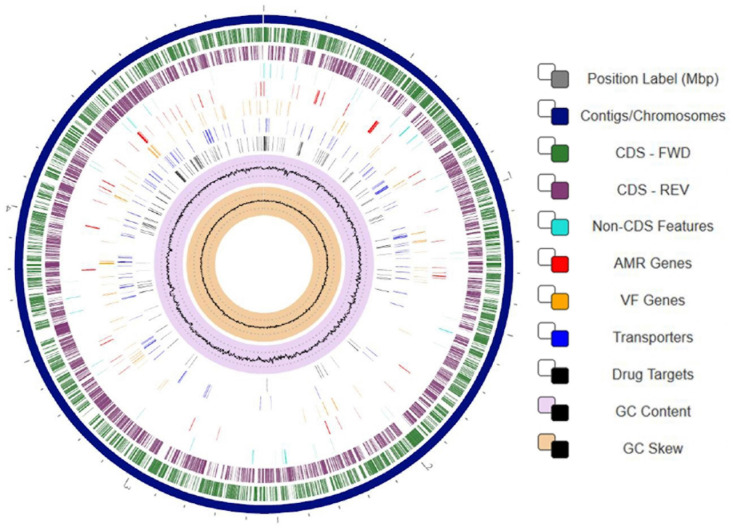
Circular visualization of the *Rouxiella badensis* strain SER3 genome. Numbers represent megabases (Mbp). From outside to the center: 47 contigs were assembled to form a scaffold representing the chromosome (navy blue track). Genes on forward strand (green), genes on reverse strand (purple), non-CDS features (turquoise), resistance genes (red), virulence factor genes (orange), transporters (blue), drug targets (black), GC content (lilac and black), and GC skew (khaki and black). To display the circular viewer, we used genome annotation service from PATRIC (RAST tool kit).

To gain better understanding of the potential traits of strain SER3 involved in postharvest phytopathogen biocontrol, its genome was sequenced. The SER3 genome consisted of 47 contigs, and the quality of the assembly was evaluated with Quast^4^, with approximately 5.08 Mb, a GC content of 52.8%, and 4,545 open reading frames, among other genes that code for ribosomal genes (Table 2 and Figure 6). Similar numbers are also found in other *Rouxiella* genomes. The genome sequences were deposited in GenBank under the following accession numbers: NZ_CP049603.1; BioProject, PRJNA224116; and BioSample, SAMN14066751.

**TABLE 2:** Genome characteristics of strain SER3.

Size (Mb)	5.08
GC%	52.8%
Protein	4,545
rRNAs	4
tRNAs	61
Other RNA	6
Gene	4,684
Pseudogene	69
Scaffolds	1
Contigs	47
N50	255,898
L50	8

### Taxonomic Affiliation of SER3

**FIGURE 7:**
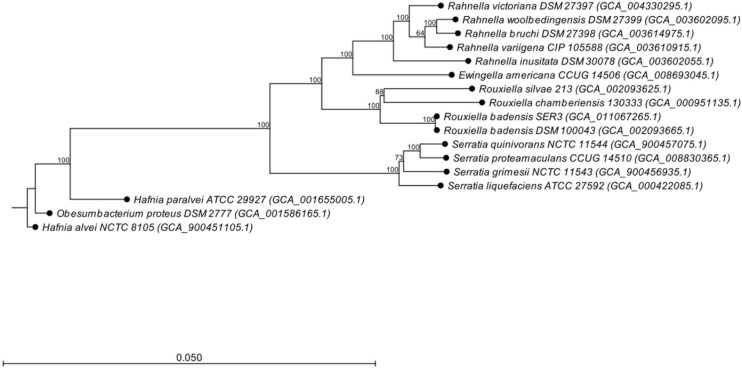
Phylogenomic tree based on the whole genome sequence data of *Rouxiella badensis* strain SER3, including the relationship with other bacterial species. The phylogenetic tree was constructed using the neighbor-joining algorithm. Bootstrap analysis of 1,000 replications was performed and is expressed as a percentage. *Hafnia alvei* NCTC 8,105 was used as an outgroup.

Based on the sequences of the 16S rRNA gene, SER3 showed 100% identity with the type strains of *R. badensis* DSM 100043^T^ (Supplementary Figure 3). A phylogenomic approach confirmed the close relationship with the *R. badensis* DSM 100043 type strain (Figure 7). Moreover, a comparison at the genomic level of strain SER3 through ANI > 95–96% and the GGDC > 70% also showed that it is strongly affiliated with *R. badensis* (Table 3).

**TABLE 3:** OGRIs values obtained from the genome comparison of strain SER3 and closely related species.

**Species/Strain**	**16S ≥98.7%**	**ANI ≥96%**	**GGDC ≥70%**
*Rouxiella badensis* DSM 100043^T^	100	99.69	98.20
*Rahnella variigena* CIP 105588^T^	99.51	76.41	21.10
*Obesumbacterium proteus* DSM 2777^T^	99.31	73.14	21.30
*Hafnia paralvei* ATCC 29927^T^	99.31	72.56	20.40
*Rouxiella chamberiensis* 130333^T^	99.31	80.56	23.80
*Rahnella bruchi* DSM 27398^T^	99.31	76.23	20.90
*Rahnella woolbedingensis* DSM 27399^T^	99.31	76.28	21.00
*Rahnella inusitata* DSM 30078^T^	99.21	76.47	20.80
*Rouxiella silvae* 213^T^	99.21	80.88	24.00
*Serratia liquefaciens* ATCC 27592^T^	99.12	75.04	20.60
*Serratia grimesii* NBRC 13537^T^	99.12	73.94	20.40
*Ewingella americana* ATCC 33852^T^	99.12	76.81	21.30
*Serratia proteamaculans* CCUG 14510^T^	99.12	74.66	20.20
*Serratia quinivorans* NCTC 11544^T^	99.03	74.81	20.30
*Rahnella victoriana* DSM 27397^T^	98.98	76.56	21.10
*Hafnia alvei* ATCC 13337^T^	98.72	72.74	21.10

### Search for Biocontrol Gene Clusters in SER3 and Related Genomes

The antiSMASH program was used to determine the potential compounds involved in the postharvest biocontrol of phytopathogens by *R. badensis* SER3 and other closely related species, including two *R. badensis* strains (DSM 100043 and WG36), *Rhanella* spp., and *Serratia* spp. In the SER3 genome, biosynthetic gene clusters involved in siderophores (100%) and polyenes (77%) were observed. In addition, compounds such as thiopeptides, non-ribosomal peptide synthetases (NRPS), and polyketide synthases (PKS) were identified (Table 4). Similar biosynthetic clusters and percentages were observed in the other two *R. badensis* genomes analyzed, corroborating their close phylogenomic similarity. A 100% similarity was observed for siderophore biosynthetic clusters in *Rhanella* spp. and *Ewingella americana* CCUG 14506, and in *Obesumbacterium proteus* DSM 2777 and *Hafnia* spp. with a good similarity score (75%) in their respective genomes.

**TABLE 4:** AntiSMASH analysis and prediction of biosynthetic compounds in *R. badensis* SER3 and related bacterial genomes.

**Bacterial species/strain**	**NRPS**	**Sidero phore**	**Thiopeptide**	**Arylpolyene**	**T1PKS**	**T3PKS**	**transAT-PKS**	**Hserlac tone**	**Redox-cofactor**	**transAT-PKS-like**	**thioamitides**	**Nrps-like**	**Terpene**	**Betalactone**	**RRE-containing**	**Ladderane**	**RiPP-like**	**Pyrrolnitrin**
*Rouxiella badensis* SER3	38%	100%	14%	77%	+	18%	40%	+	13%	–	–	–	–	–	–	–	–	–
*Rouxiella badensis* DSM 100043	38%	100%	14%	72%	+	18%	40%	–	13%	–	–	–	–	–	–	–	–	–
*Rouxiella badensis* WG36	23%	100%	+	77%	+	12%	+	–	13%	+	–	–	–	–	–	–	–	–
*Rouxiella silvae* 213	–	–	14%	77%	–	–	–	–	+	–	+	20%	–	–	–	–	–	–
*Rouxiella silvae* Leaf50	38%	–	14%	77%	–	–	–	+	+	–	+	–	–	–	–	–	–	–
*Rouxiella chamberiensis* 130333	38%	–	14%	–	–	–	40%	+	+	–	–	–	100%	–	–	–	–	–
*Rahnella bruchi* DSM 27398	+	100%	14%	77%	–	–	–	+	–	–	–	–	–	+	13%	+	–	–
*Rahnella woolbedingensis* DSM 27399	–	100%	14%	77%	–	–	–	–	–	–	–	–	–	+	+	–	–	–
*Rahnella inusitata* DSM 30078	38%	100%	14%	66%	–	–	–	–	–	–	–	–	–	+	–	–	–	–
*Rahnella variigena* CIP 105588	–	100%	14%	72%	–	–	–	+	–	–	–	–	–	+	13%	–	–	–
*Rahnella Victoriana* DSM 27397	–	100%	14%	77%	–	–	–	2%	–	–	–	–	–	–	13%	–	–	–
*Ewingella americana* CCUG 14506	–	100%	14%	77%	–	–	–	–	–	–	–	–	–	+	–	–	+	–
*Serratia liquefaciens* ATCC 27592	57%	+	14%	83%	–	–	–	–	–	–	–	+	–	+	–	–	–	–
*Serratia proteamaculans* CCUG 14510	57%	+	–	73%	–	–	–	+	–	–	–	–	75%	+	–	–	–	–
*Serratia quinivorans* NCTC 11544	57%	+	+	77%	–	–	–	+	–	–	–	80%	–	+	–	–	–	–
*Serratia grimesii* NCTC 11543	57%	+	14%	77%	+	–	–	–	–	–	–	–	–	+	–	–	+	100%
*Obesumbacterium proteus* DSM 2777	–	75%	14%	–	–	–	–	+	–	–	–	–	–	+	–	–	–	–
*Hafnia paralvei* ATCC 29927	–	75%	14%	–	–	–	–	6%	–	–	–	–	–	+	–	–	–	–
*Hafnia alvei* NCTC 8105	–	75%	14%	+	–	–	–	+	–	–	–	–	–	+	–	–	–	–

## Discussion

Berries (strawberries, blackberries, and blueberries) have a very short shelf life after harvest. Therefore, they must be immediately distributed for use, preferably under cold chain, which considerably hinders their international commercialization. High postharvest fruit losses due to phytopathogenic fungal diseases are related to high humidity levels, increased nutrients, low pH values, and low intrinsic resistance to postharvest decomposition and fungal diseases (Dukare et al., 2019). During the decomposition process, the fruit loses weight and decreases in firmness and quality, resulting in economic losses. In many instances, synthetic chemical compounds are used as coatings to avoid pathogen infections and extend their shelf life; however, toxic residues can be hazardous to human health, in addition to restricting the global commercialization of berries. Therefore, it is important to describe the phytopathogens that affect postharvest berries as well as to develop sustainable alternatives for their biological control (Abeer et al., 2013).

Here, 20 fungal strains were isolated from berries and characterized by ITS sequencing and homology searches. They showed similarity to *Botrytis* or *Fusarium*, among others. Previous reports have shown that several of these genera are phytopathogens that cause pre- and postharvest diseases in various crops, including strawberries (Dukare et al., 2019). In particular, *B. cinerea* can easily infect berries such as strawberry, blueberry, blackberry, raspberry, cranberry, and bilberry fruit, causing drastic losses after harvest (Leroux et al., 2002; Romanazzi and Feliziani, 2014; Petrasch et al., 2019). Another type of ascomycete fungus that causes damage to various crops is *Fusarium*, which is best known for affecting the roots and some aerial parts of plants, such as stems, and causes vascular browning, leaf epinasty, stunting, progressive wilting, defoliation, floral damage, and subsequent plant death (Dean et al., 2012). Herein, two strains with highly similar identity (98.3 and 99.2%) to *F. brachygibbosum* were isolated from strawberry fruit (Table 1). To our knowledge, *F. brachygibbosum* has not been reported as a fruit phytopathogen; therefore, this would be the first report as a postharvest pathogen in fruit such as strawberries. Further studies on the morphology of *F. brachygibbosum* and analysis of other molecular markers are being conducted by our research group to corroborate this hypothesis.

Other fungal genera found in berries were *Alternaria*, *Cladosporium*, *Geotrichum*, *Mucor*, and *Penicillium*, which have already been reported as causative agents of postharvest disease in these fruit (Koike et al., 2003; Tournas and Katsoudas, 2005; Gordon et al., 2016; López et al., 2016; Pastrana et al., 2017; Petrasch et al., 2019). It should be noted that fungi belonging to beneficial species such as *Trichoderma* have also been found in berries (Santoyo et al., 2021). In the present work, the strain *Trichoderma* sp. AF4 did not produce any apparent damage when reinoculated in strawberries and showed similar results as the uninoculated controls. Moreover, preliminary studies performed in our laboratory suggest that AF4 restricts the growth of some postharvest berry pathogens.

In agreement with the aforementioned studies, the fruit microbiome has been reported to contain not only pathogenic species but also microorganisms that can naturally help fight postharvest diseases, thus reducing losses through increased shelf life and fruit quality (Droby and Wisniewski, 2018). Consequently, we isolated the SER3 strain from the surface of a strawberry fruit, and it showed antifungal activity against *Fusarium* (Supplementary Figure 1). Furthermore, during activity evaluation, SER3 exhibited significant antagonism against the postharvest pathogens isolated herein. Moreover, the volatile compounds of SER3 also exhibited inhibition of mycelial growth, although to a lesser extent, with significant inhibition being observed only against two species, viz., *P. expansum* and *F. brachygibbosum*. These results suggested that SER3 antagonizes the phytopathogens through the action of diffusible (mainly) and volatile compounds, which is consistent with other studies showing similar mechanisms of action in other bacterial species (Hernández-León et al., 2015; Wallace et al., 2017). The inhibition of mycelial growth of postharvest phytopathogens was corroborated by *in vivo* tests on strawberry fruit using *B. cinerea* and *F. brachygibbosum*. Following the coinoculation of the SER3 strain and *B. cinerea* or *F. brachygibbosum*, the hyphae presented deformations and protrusions on the surface. This type of damage in the fungal pathogen hyphae has been observed in other studies and is associated with a reduction in fungal pathogenicity (Wallace et al., 2017; Emanuel et al., 2020).

Given the relevant biocontrol properties of strain SER3, its genome was sequenced, and its taxonomic affiliation was assigned based on ANI and GGDC. Based on these parameters, SER3 was established to belong to the *R. badensis* species. *R. badensis* is a relatively new species described in 2017; it is a Gram-negative bacillus that forms whitish colonies, can grow optimally at 37°C, reduces nitrates, and produces acid from different sugars (Le Fléche-Matéos et al., 2017). To investigate the possible antifungal mechanism of *R. badensis* SER3, its genome was analyzed using the antiSMASH server (Blin et al., 2017), which led to the prediction of various antibiotic compounds and antifungal compounds such as siderophores, NRPS, and PKS. These three compounds are extracellular and are produced by a wide range of biocontrol bacterial species, such as *Bacillus* and *Pseudomonas*, and close relatives of *R. badensis*, such as *Rahnella aquatilis*, which have been characterized as antifungals (Calvo et al., 2007; Chen et al., 2007; Santoyo et al., 2012; Carmona-Hernandez et al., 2019). Likewise, NRPS and PKS are not exclusive to bacterial strains but can also be synthesized by phytopathogenic and beneficial fungi, such as *Trichoderma* (Mukherjee et al., 2012). Other compounds reported to have been found in the *R. badensis* SER3 genome were a cluster of desferrioxamine-type siderophores (100% similarity), which are iron-chelating compounds (Boiteau et al., 2019) and can restrict the growth of pathogens (Kloepper et al., 1980; de los Santos-Villalobos et al., 2012). They have been reported in a wide range of biocontrol and plant growth promoter species (Crowley, 2006; Wang et al., 2020). Interestingly, the same compounds, such as siderophores, NRPS, and arylpolyene compounds, with similar percentages of identity were detected using antiSMASH in two other *R. badesis* genomes. Similarly, close relatives of *R. badensis*, such as *Rahnella*, also presented good similarity percentages with clusters for the synthesis of siderophores, thiopeptides, and arylpolyenes in their respective genomes. Other bacterial species, including those belonging to genera such as *Ewingella* (Roy Chowdhury et al., 2007), *Obesumbacterium* (Amin et al., 2014), and *Hafnia*, contain highly similar clusters for the synthesis of potential compounds, such as siderophores (100%). These results support the proven role of SER3 in the biocontrol of fungal pathogens and similar roles reported by Calvo et al. (2007) and Chen et al. (2007) in *Rahnella* and *Serratia* genera. To our knowledge, the potential role in the biocontrol of plant fungal pathogens has not been described for the rest of the bacterial species analyzed here with antiSMASH (Table 4).

## Conclusion

In this study, a functional analysis of the biocontrol activities of the novel strain SER3 against postharvest pathogenic fungi of berries was performed, which showed a high genomic and phylogenetic identity with *R. badensis*. Thus, we propose a new ecological role for this species and other species of the genus *Rouxiella*. Notably, SER3 genome provides some indications of the antifungal modes of action; however, other mechanisms of biocontrol by *R. badensis* SER3 cannot be excluded, since other antifungal activities, such as the activity of lytic enzymes, have not been explored. In addition, antiSMASH analysis for other species analyzed in this study provides some clues of possible antagonistic action toward plant pathogens, although this hypothesis requires further investigation. Lastly, isolation of SER3 presents a new option in the biocontrol of postharvest pathogens of berries and provides new opportunities to investigate its role as a promoter of plant growth through direct mechanisms.

## Data Availability Statement

The datasets presented in this study can be found in online repositories. The names of the repository/repositories and accession number(s) can be found below: https://www.ncbi.nlm.nih.gov/genbank/, NZ_CP049603.1.

## Author Contributions

LRM-C conducted the experiments, analyzed the data, and prepared the figures and tables. SS-V and GS conceived and designed the experiments and analyzed the data. GS wrote the first draft of the manuscript. All authors contributed to manuscript revision, read, and approved the submitted version.

## Conflict of Interest

The authors declare that the research was conducted in the absence of any commercial or financial relationships that could be construed as a potential conflict of interest.

## Publisher’s Note

All claims expressed in this article are solely those of the authors and do not necessarily represent those of their affiliated organizations, or those of the publisher, the editors and the reviewers. Any product that may be evaluated in this article, or claim that may be made by its manufacturer, is not guaranteed or endorsed by the publisher.

## Footnotes

1
https://www.patricbrc.org/

2
http://rast.theseed.org/FIG/rast.cgi

3
http://rast.nmpdr.org

4
http://quast.sourceforge.net/quast

## Supplementary Material

Supplementary Figure 1 Effect of diffusible compounds of SER3 following direct co-inoculation with the Fusarium pathogen. The bacterial strain was streaked onto plates in a cross shape, and mycelial plugs 4 mm in diameter were deposited at the center of the quadrants formed. Experiments were independently performed a minimum of three times. The plates were incubated, and mycelial growth was measured on day 3. The percentage of growth inhibition was measured as follows:% growth inhibition = [(Ac – Ab)/Ac] × 100, where Ac is the control mycelial area, and Ab is the mycelial area with treatment.

Supplementary Figure 2 Koch’s postulates. Berries were infected (*n* = 18) with spore solutions (1 × 10^5^ spores/mL). Panel **(A)** shows blueberries inoculated with *Cladosporium sp*. 1BOA spores, while panel **(B)** shows strawberries inoculated with *Penicillium expansum* 230 spores, and panel **(C)** shows strawberries inoculated with *Mucor circinelloides* 1BF spores.

Supplementary Figure 3 Phylogenetic tree based on the 16S ribosomal gene sequence of *Rouxiella badensis* strain SER3, including the relationship with other bacterial species (nucleotide sequence can be accessed in GenBank: CP049603). A phylogenetic tree was constructed using the maximum-likelihood algorithm. Bootstrap analysis of 1000 replications was performed and expressed as a percentage, and the most common enterobacteria were used as an outgroup.
