# Supplementary figures and images for: Functional and Genomic Analysis of Rouxiella badensis SER3 as a Novel Biocontrol Agent of Fungal Pathogens
Source: Front Microbiol. 2021 Aug 5;12:709855. doi: 10.3389/fmicb.2021.709855 (PMC8375033; doi:10.3389/fmicb.2021.709855)

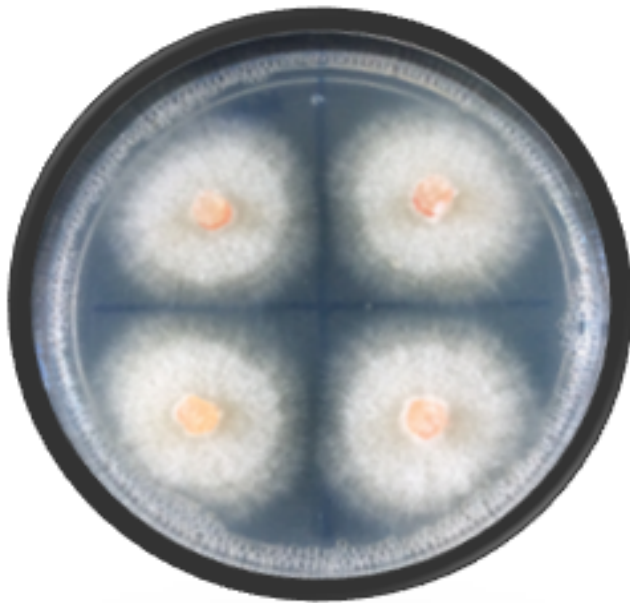

*Fusarium brachygibbosum* 4BF

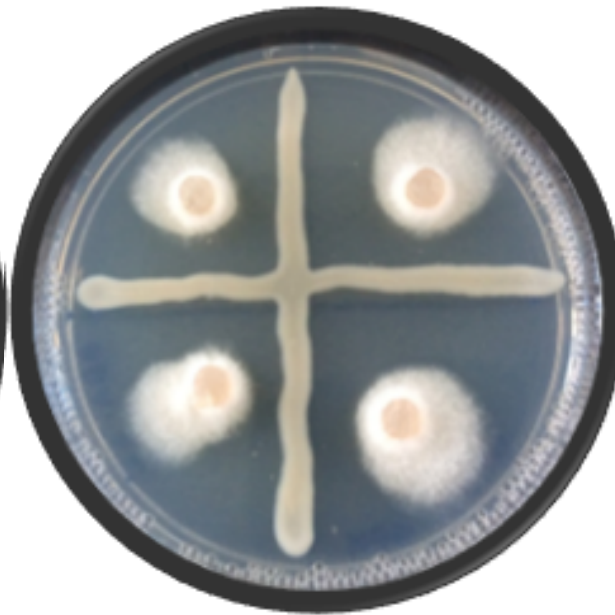

*Fusarium brachygibbosum* 4BF

+

SER3 strain

Supplement: Supplementary Figure 1 — Effect of diffusible compounds of SER3 following direct co-inoculation with the Fusarium pathogen. The bacterial strain was streaked onto plates in a cross shape, and mycelial plugs 4 mm in diameter were deposited at the center of the quadrants formed. Experiments were independently performed a minimum of three times. The plates were incubated, and mycelial growth was measured on day 3. The percentage of growth inhibition was measured as follows:% growth inhibition = [(Ac – Ab)/Ac] × 100, where Ac is the control mycelial area, and Ab is the mycelial area with treatment. [file Data_Sheet_1.PDF]

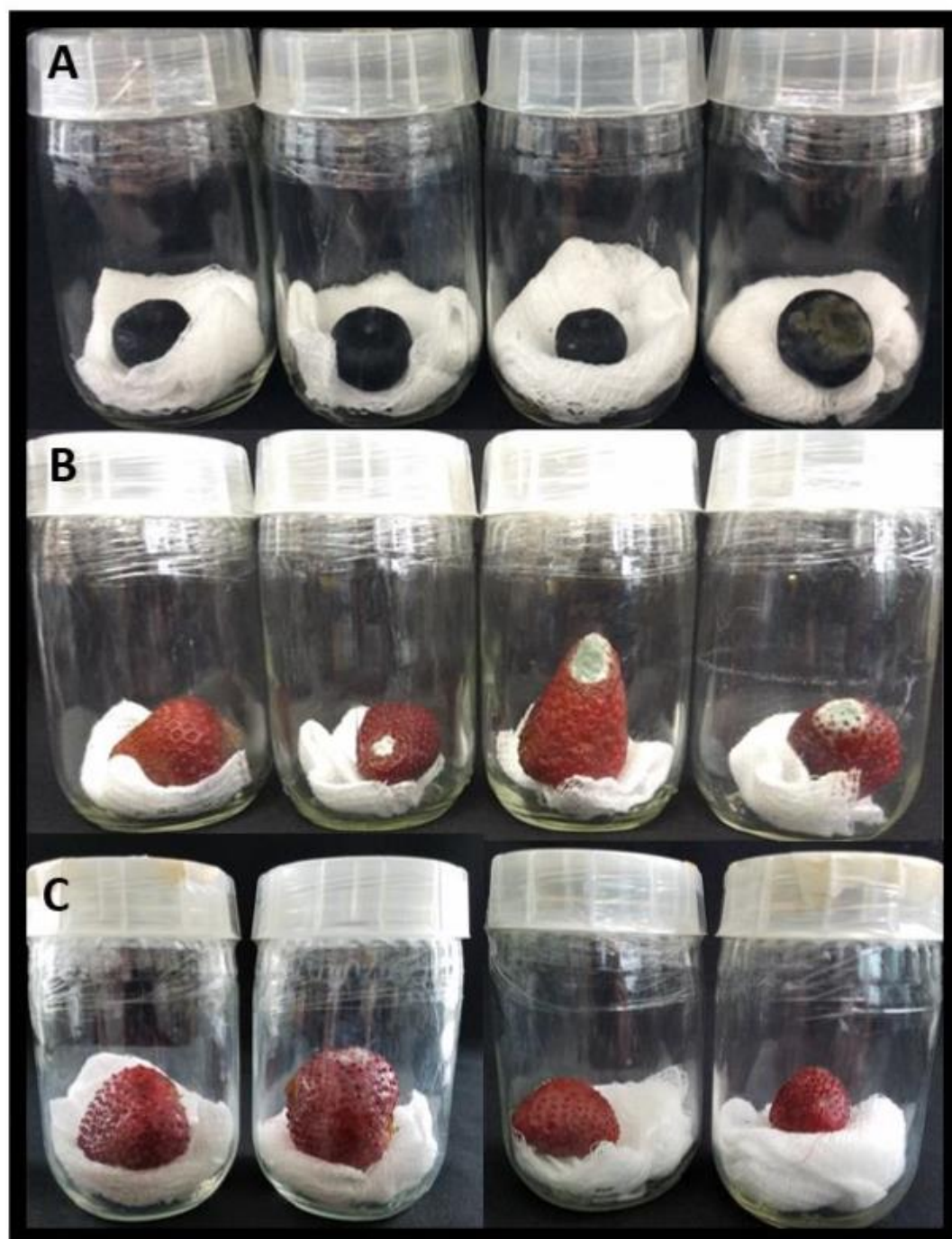

Supplement: Supplementary Figure 2 — Koch’s postulates. Berries were infected (n = 18) with spore solutions (1 × 10^5^ spores/mL). Panel (A) shows blueberries inoculated with Cladosporium sp. 1BOA spores, while panel (B) shows strawberries inoculated with Penicillium expansum 230 spores, and panel (C) shows strawberries inoculated with Mucor circinelloides 1BF spores. [file Data_Sheet_2.PDF]

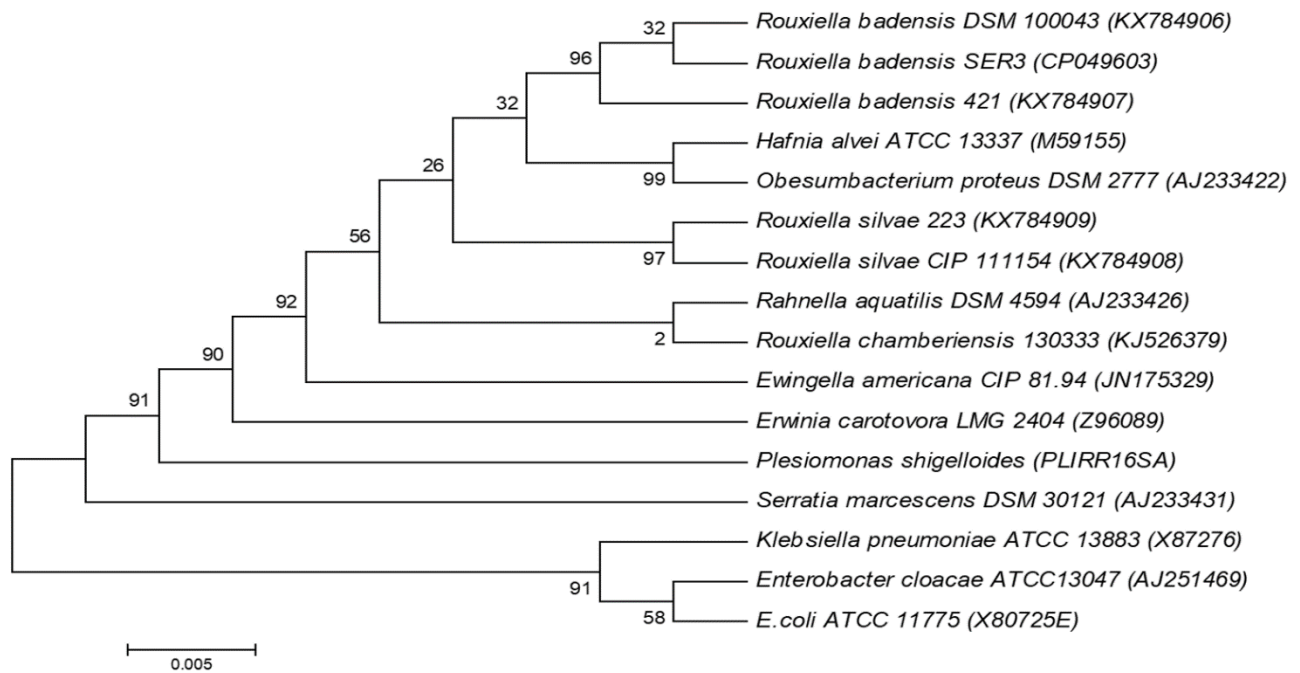

Supplement: Supplementary Figure 3 — Phylogenetic tree based on the 16S ribosomal gene sequence of Rouxiella badensis strain SER3, including the relationship with other bacterial species (nucleotide sequence can be accessed in GenBank: CP049603). A phylogenetic tree was constructed using the maximum-likelihood algorithm. Bootstrap analysis of 1000 replications was performed and expressed as a percentage, and the most common enterobacteria were used as an outgroup. [file Data_Sheet_3.PDF]
